# Multiomics modeling of the immunome, transcriptome, microbiome, proteome and metabolome adaptations during human pregnancy

**DOI:** 10.1093/bioinformatics/bty537

**Published:** 2018-07-02

**Authors:** Mohammad Sajjad Ghaemi, Daniel B DiGiulio, Kévin Contrepois, Benjamin Callahan, Thuy T M Ngo, Brittany Lee-McMullen, Benoit Lehallier, Anna Robaczewska, David Mcilwain, Yael Rosenberg-Hasson, Ronald J Wong, Cecele Quaintance, Anthony Culos, Natalie Stanley, Athena Tanada, Amy Tsai, Dyani Gaudilliere, Edward Ganio, Xiaoyuan Han, Kazuo Ando, Leslie McNeil, Martha Tingle, Paul Wise, Ivana Maric, Marina Sirota, Tony Wyss-Coray, Virginia D Winn, Maurice L Druzin, Ronald Gibbs, Gary L Darmstadt, David B Lewis, Vahid Partovi Nia, Bruno Agard, Robert Tibshirani, Garry Nolan, Michael P Snyder, David A Relman, Stephen R Quake, Gary M Shaw, David K Stevenson, Martin S Angst, Brice Gaudilliere, Nima Aghaeepour

**Affiliations:** 1 Department of Anesthesiology, Perioperative and Pain Medicine, Stanford University School of Medicine, Stanford, CA, USA; 2 Département de Mathématiques et de Génie Industriel, École Polytechnique de Montréal, QC, Canada; 3 Groupe d’Études et de Recherche en Analyse des Décision (GERAD), Montréal, QC, Canada; 4 Centre Interuniversitaire de Recherche sur les Réseaux d’Entreprise, la Logistique et le Transport (CIRRELT), Montréal, QC, Canada; 5 Department of Medicine, Stanford University School of Medicine, Stanford, CA, USA; 6 Veterans Affairs Palo Alto Health Care System, Palo Alto, CA, USA; 7 Department of Genetics, Stanford University School of Medicine, Stanford, CA, USA; 8 Department of Population Health and Pathobiology, College of Veterinary Medicine, North Carolina State University, Raleigh, NC, USA; 9 Department of Bioengineering, Stanford University, Stanford, CA, USA; 10 Cancer Early Detection Advanced Research Center, Knight Cancer Institute and Department of Molecular and Medical Genetics, Oregon Health Sciences University, Portland, OR, USA; 11 Department of Neurology and Neurological Sciences, Stanford University School of Medicine, Stanford, CA, USA; 12 Department of Microbiology and Immunology, Stanford University, Stanford, CA, USA; 13 Institute for Immunity, Transplantation and Infection, Human Immune Monitoring Center Stanford, CA, USA; 14 Division of Neonatology, Department of Pediatrics, Stanford University School of Medicine, Stanford, CA, USA; 15 Institute for Computational Health Sciences, University of California San Francisco, San Francisco, CA, USA; 16 Department of Pediatrics, University of California San Francisco, San Francisco, CA, USA; 17 Department of Obstetrics and Gynecology, Stanford University School of Medicine, Stanford, CA, USA; 18 Departments of Biomedical Data Sciences and Statistics, Stanford University, Stanford, CA, USA; 19 Department of Statistics, Stanford University School of Medicine, Stanford, CA, USA

## Abstract

**Motivation:**

Multiple biological clocks govern a healthy pregnancy. These biological mechanisms produce immunologic, metabolomic, proteomic, genomic and microbiomic adaptations during the course of pregnancy. Modeling the chronology of these adaptations during full-term pregnancy provides the frameworks for future studies examining deviations implicated in pregnancy-related pathologies including preterm birth and preeclampsia.

**Results:**

We performed a multiomics analysis of 51 samples from 17 pregnant women, delivering at term. The datasets included measurements from the immunome, transcriptome, microbiome, proteome and metabolome of samples obtained simultaneously from the same patients. Multivariate predictive modeling using the Elastic Net (EN) algorithm was used to measure the ability of each dataset to predict gestational age. Using stacked generalization, these datasets were combined into a single model. This model not only significantly increased predictive power by combining all datasets, but also revealed novel interactions between different biological modalities. Future work includes expansion of the cohort to preterm-enriched populations and in vivo analysis of immune-modulating interventions based on the mechanisms identified.

**Availability and implementation:**

Datasets and scripts for reproduction of results are available through: https://nalab.stanford.edu/multiomics-pregnancy/.

**Supplementary information:**

Supplementary data are available at *Bioinformatics* online.

## 1 Introduction

Physiological changes during pregnancy are highly dynamic and involve coordinated changes among multiple interconnected molecular and cellular systems from the fetus, the fetal-membrane and the mother (Diemert and Arck, 2018; Menon *et al.*, 2016). The simultaneous interrogation of these systems can reveal otherwise unrecognized crosstalk. Understanding such crosstalk can inform several lines of investigation. From a biological perspective, it can point to important disease mechanisms such as immune programming by the microbiome, or specific interactions between proteins and cellular elements (Aghaeepour *et al.*, 2017; Dethlefsen *et al.*, 2007). From a diagnostic perspective, it can reveal biomarkers from several biological domains that provide higher predictive power if combined. Alternatively, it can point to alternative biomarkers in an accessible biological compartment, which can replace biomarkers that are difficult to obtain or expensive to measure.

Recent technological advances in science provide novel opportunities to unravel the complex biology of pregnancy. A particularly pressing issue is to identify the biological pathways and the converging pathological processes that lead to preterm birth (Lackritz *et al.*, 2013). Preterm birth is the major cause of neonatal death, and the second leading cause of mortality in children under the age of 5 years (Liu *et al.*, 2012). An ongoing cohort study by the March of Dimes Prematurity Research Center at Stanford University exploits recent technological advances to examine an array of biological, demographic, clinical and environmental factors associated with normal and pathological pregnancies (Stevenson *et al.*, 2013; Shaw *et al.*, 2018; Wise *et al.*, 2017). From a biological perspective, this effort has so far produced two major lines of evidence. One line sheds light onto precisely tuned chronological changes that occur during normal pregnancy. For example, a highly multiplexed cell-based assay in whole blood revealed an ‘immunological clock’ of human pregnancy that predicts gestational age at the time of sampling (Aghaeepour *et al.*, 2017). Similar results were reported in a longitudinal analysis of cell-free, maternal RNA (Pan *et al.*, 2017) and plasma proteins (Aghaeepour *et al.*, 2018). The primary objective of using gestational age as the clinical outcome in these studies is to extract molecular features that best capture normal chronological changes over the course of term pregnancy. Such knowledge will elucidate molecular deviations that are associated with pregnancy-related pathologies. The second line of this work points to important pathophysiological derangements. For example, dense longitudinal sampling of the vaginal microbiome revealed community composition profiles associated with preterm birth that were validated in an independent cohort (Callahan *et al.*, 2017; DiGiulio *et al.*, 2015). However, the important work of bringing these data modalities together has remained unexplored.

From a bioinformatics point of view, current multiomics efforts belong to two categories generally known as multi-staged and meta-dimensional (Ritchie *et al.*, 2015; Rohart *et al.*, 2017). In multi-staged analyses, measurements of the same biological factors (e.g. genes) are integrated at various biological levels and using different technological platforms (e.g. DNA and RNA sequencing, epigenetic analysis and proteomics assays—notable examples include (Emilsson *et al.*, 2008; Maynard *et al.*, 2008; Schadt *et al.*, 2005; Shabalin, 2012; Shen *et al.*, 2009)). However, recent biological studies extend well beyond just measurements of the same gene/protein and include various assays that cannot be mapped to a single gene. These include single cell analysis (Aghaeepour *et al.*, 2017), imaging (Woodward *et al.*, 2006), profile of metabolic profiling (Piening *et al.*, 2018), actigraphy using wearable sensors (Halilaj *et al.*, 2018) and clinical phenotypes (Ferrero *et al.*, 2016). Meta-dimensional multiomics approaches are now emerging that aim to combine heterogeneous datasets to identify key factors at various biological levels, their interactions with each other, and with clinical outcomes. Some studies achieve this by simply merging all available datasets into a single matrix for joint modeling (Fridley *et al.*, 2012; Holzinger *et al.*, 2014; Mankoo *et al.*, 2011). These approaches are often susceptible to biases introduced by the differential sizes, modularities, scalings and batch effects of the included datasets. Various kernel (e.g. Borgwardt *et al.*, 2005) and graph (e.g. Kim *et al.*, 2012) transformations as well as latent space projections (Singh *et al.*, 2016) have been proposed to address these biases. In settings where analysis is performed against an external factor, an alternative is to use mixture-of-experts methods to combine the results of independent models produced using each dataset through various algorithms ranging from voting (e.g. Aghaeepour and Hoos, 2013) to integration of posterior Bayesian probabilities (Akavia *et al.*, 2010; Zhu *et al.*, 2008, 2012).

The main objective of this study was to test multiple strategies for integrating transcriptomic, immunological, microbiomic, metabolomic and proteomic datasets into different statistical models predicting gestational age in term pregnancy and identify the most accurate strategy. A final objective was to interrogate the derived model for novel and testable biological hypothesis.

## 2 Materials and methods

### 2.1 Study design

Pregnant women presenting to the obstetrics clinics of the Lucile Packard Children’s Hospital at Stanford University for prenatal care were invited to participate in a cohort study to prospectively examine environmental and biological factors associated with normal and pathological pregnancies. Women were eligible if they were at least 18 years of age and in their first trimester of a singleton pregnancy. In 17 women, three samples were collected during pregnancy and a fourth one after deliver. The time points were chosen such that a peripheral blood sample (CyTOF analysis), a plasma sample (proteomic, cell-free transcriptomics, metabolomics analyses), a serum sample (luminex analyses) and a series of culture swabs (microbiome analysis) were simultaneously collected from each woman during the first (7–14 weeks), second (15–20 weeks) and third (24–32 weeks) trimester of pregnancy and 6-week postpartum. Repeated sampling during pregnancy allowed assessing important biological adaptations occurring continuously from the early phases of fetal development (first trimester) to the late phases of gestation (third trimester). The sample collected 6-week postpartum allowed for the assessment of the biological variables after the delivery of the fetus, a surrogate for the non-pregnant state which is not accessible in a prospective study of pregnant women.

### 2.2 Gestational age estimation

Gestational age was determined by best obstetrical estimate as recommended by the American College of Obstetricians and Gynecologists (Hershey, 2014).

### 2.3 Biological assays

Plasma and serum samples were assayed using the Luminex platform for cytokine levels. In addition, plasma samples were used for proteomics analysis, LC-MS metabolomics analysis, and cell-free transcriptomic analysis. Whole blood samples were analyzed using mass cytometry for single-cell characterization of the immune system. Finally, vaginal swabs, stool, saliva and tooth/gum samples were used for microbiomic profiling. See Supplementary Material for more detailed description of the assays. All timepoints of a given patient were analyzed simultaneously by all omics platforms to minimize systematic technical confounders (Supplementary Fig. S4).

### 2.4 Multivariate modeling

For a matrix **X** of all features from a given dataset, and a vector of estimated gestational ages at the time of each sampling, **Y**, the EN algorithm calculates coefficients β to minimize the error term $L(\beta )=||Y-X\beta |{|}^{2}$. An *L*_1_ regularization (Tibshirani, 1996) was used to increase model sparsity (which facilitates biological interpretation and validation). However, this approach is not ideal for the analysis of the highly interrelated biological datasets, because it only selects representatives of communities of highly correlated features. As a result, features correlated to these selected representatives are disregarded, despite the fact that they could be biologically relevant. This limitation is addressed by using an additional *L*_2_ regularization penalty: $L(\alpha ,\lambda ,\beta )=||Y-X\beta |{|}_{2}+\lambda [(1-\alpha )||\beta |{|}_{2}+\alpha ||\beta |{|}_{1}]$, where $||\beta |{|}_{2}=\beta^{⊤}\beta$ and $\left||\beta |{|}_{1}=\sum_{i=1}^{n}|\beta_{i}\right|$. The subset selecting factor *λ* controls the sparsity of the model and the smoothing factor *α* controls the smoothing of selection from correlated variables (Zou and Hastie, 2005).

### 2.5 Stack generalization

In the computer science literature, stacked generalization refers to the practice of combining several weak predictors for increased predictive power (Breiman, 1996; Sharkey, 1996; Wolpert, 1992). In life sciences, this often translates to analysis of a single dataset using multiple algorithms and then combining the results in a final multivariate modeling step (Ge and Wong, 2008; He *et al.*, 2013; Larranaga *et al.*, 2006; Yang *et al.*, 2010; Wang *et al.*, 2006). Here we expand this concept to multiomics analysis where a single multivariate analysis algorithm (EN) is used on a cohort of patients, and the variable factor is the biological assays used for developing the datasets. First, an EN model is constructed on each dataset from the same subjects. Then, all estimations of gestational age at time of sampling are used as features for a final EN model. This, essentially, is a weighted average of the individual models where the weights are the coefficients of the EN model.

### 2.6 Cross-validation

An underlying assumption of the EN algorithm is statistical independence between all observations. In this analysis, while the subjects are independent, the samples collected from various trimesters of the same subject are not. To account for this, we designed a leave-one-subject-out cross-validation strategy. In this setting, a model is trained on all available samples except for the three trimesters of a given subject. The model is then tested on all samples of the subject that it was blinded to. This process is repeated for all subjects until a blinded prediction has been produced for all samples. Final results are reported using these blinded predictions. This ensures complete independence from any intra-subject correlations.

A two-layer cross-validation strategy was implemented for simultaneous free-parameter optimization and analysis of the generalizability of the results (Fig. 2A). The inner layer selects the best values of *α* and *λ* (see Supplementary Fig. S1). The outer layer ensures that performance is reported on subjects that the models were blinded to during training.

A similar strategy was used for the stacked generalization step. Cross-validation folds where synchronized between the individual models from each dataset and the integrated model to leave out the same set of data points at all levels of the analysis. Importantly, this guarantees that not only the stacked generalization model, but also its input features (i.e. the final predictions from each dataset) were blinded to the same subject during cross-validation.

### 2.7 Empirical evaluation

The procedure described above was empirically compared against a number of standard multivariate algorithms. The same algorithms were used for the individual datasets as well stacked generalization (Fig. 5). The algorithms included Random Forest (Breiman, 2001), Gaussian Process (Williams and Barber, 1998), Support Vector Regression (Chang and Lin, 2011; Hsu and Lin, 2002) and XGboost (Chen and Guestrin, 2016). The algorithms were compared using the default implementations provided in the following packages: (Chen and He, 2015; Karatzoglou *et al.*, 2004; Liaw and Wiener, 2002). All algorithms were evaluated using the same two-layer leave-one-patient-out CV strategy. The cross-validated parameter space for Gaussian process and Support Vector Regression included all available kernels [as described in (Karatzoglou *et al.*, (2004)] and initial noise variance between 0.001 and 10 000. EN predominantly outperforms the other methods on most datasets, followed by support vector regression. XGboost outperforms the other algorithms on the microbiome dataset.

### 2.8 Model reduction

A bootstrapping procedure was used to reduce the number of features used in each model. As described in Aghaeepour *et al.* (2017), one hundred bootstrap iterations were performed on each dataset where 57 samples were drawn randomly and with replacement. Piece-wise regression between the number of features (calculated by applying a range of thresholds to the mean coefficient of each measurement across all bootstrap iterations) and the final results of the models were used to select the number of features for each modality (Oosterbaan, 1994).

### 2.9 Correlation network

The features from the reduced models were visualized using a graph structure. Each feature was represented by a node. The correlation structure between the features was extracted using a Minimum Spanning Tree (MST) where the width of the edges were proportional to the spearman *P*-value of the correlation between the two nodes, on a ${\;\text{log}\;}_{1}0$ scale. The graph was visualized using the Fruchterman-Reingolds layout (Fruchterman and Reingold, 1991).

### 2.10 *P*-value adjustment

All *P*-values were adjusted using Bonferroni’s method (adjusted-P-value=min{1,raw-P-value×n}), where *n* is the number of features (Dunn, 1961).

### 2.11 Missing value interpolation

Missing values for all datasets were interpolated using a non-parametric multivariate model based on random forests. A model was trained for each feature of each dataset, and was subsequently used to estimate the missing values as described in Stekhoven and Bühlmann (2012).

## 3 Results

### 3.1 Modularity and size

**Fig. 1.**
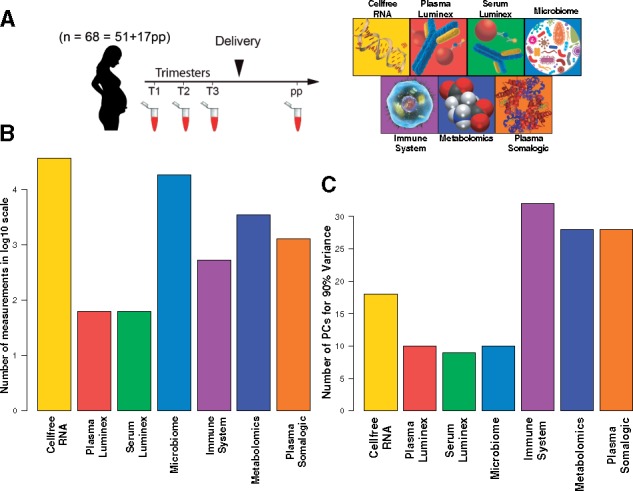
(**A**) Overview of the study design. A total of 357 samples from 51 visits by 17 women were collected during three trimesters of pregnancy, as well as an additional 17 samples 6 weeks after delivery. Seven datasets were produced for each visit by each subject. (**B**) Data from each time point of each subject were analyzed using seven high-throughput assays, which produced different number of measurements. (**C**) The seven datasets had a range of correlations among the measured features. The internal correlation between features from each dataset was quantified using the number of Principle Components (PCs) needed to capture 90% variance (datasets in which most features are highly correlated would need fewer principal components)

Samples from 17 women for a total of 51 timepoints throughout pregnancy and 6 weeks postparturm were collected. Samples were analyzed for seven biological modalities: cell-free transcriptomics, antibody-based cytokine measurements in plasma and serum, microbiomic analyses (of vaginal swabs, stool, saliva and tooth/gum), mass cytometric analyses of whole blood, untargeted metabolomics and targeted proteomics analysis of plasma. These datasets produced different levels of modularity (as measured by the number of principal components needed to account for 90% variance of each dataset—Fig. 1C). The modularity of the datasets (Fig. 1C) was not correlated with the number of measurements available (Fig. 1B).

### 3.2 Per-dataset analysis

**Fig. 2.**
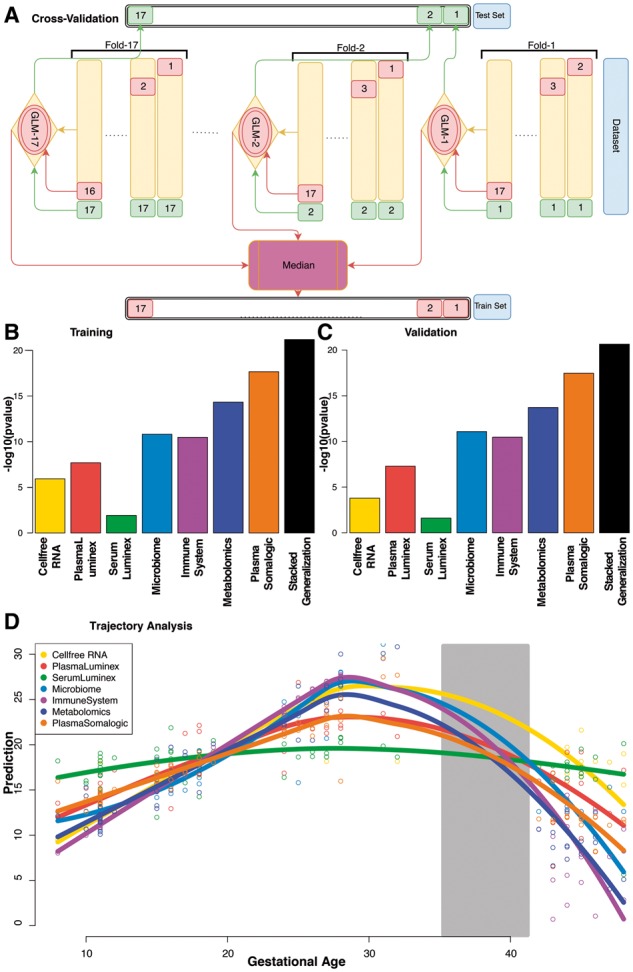
(**A**) Overview of the two-layer CV procedure. On the outer layer, a modified leave-one-out procedure is used in which all samples from the same subject (as opposed to just one sample) are left out as a blinded dataset. Within each fold, a second CV procedure is performed to optimize the free parameters of the EN model. Test samples for the inner and outer layers are visualized in red and green, respectively. The final training prediction is the median of predictions from all models that included that patient during their training (bottom), and the final blinded test set prediction comes from the only model that was blinded to it (top). See Section 2 for details. (**B**) and (**C**) The Spearman correlation *P*-values of the (B) training set and (C) test set results of the CV procedure for each dataset. (**D**) The models for each dataset applied to all samples including the postpartum visit 6 weeks after delivery. The average trend for each platform is visualized using kernel density estimation for smoothing. The delivery range is highlighted in gray. Some models quickly recover towards a non-pregnant status (below the first trimester) while others remain stable after delivery

An Elastic Net (EN) model was developed to predict the gestational age of pregnancy of each subject at each visit. A two layer Cross-Validation (CV) procedure was used to both optimize the free parameters of the EN model (see Supplementary Fig. S1) and to ensure that predictions were made on samples that were not used for training model coefficients (see Fig. 2A and Section 2). Supplementary Figure S2 visualizes the predictions on the test samples for each modality versus the clinical estimations of gestational age. *P*-values of correlation with gestational age at time of sampling for the training and testing procedures are presented in Figure 2B and C, respectively. Plasma proteomics analysis using the SomaLogic platform produced the strongest predictive power (Fig. 2B and Supplementary Fig. S6). Results remained generally consistent between training and test sets (Fig. 2C). The datasets with a higher degree of independence between features (Fig. 1C) had a higher predictive power regardless of their size.

Due to the absence of true pre-pregnancy samples, we applied these models to postpartum samples collected 6 weeks postpartum as a surrogate for a non-pregnant state. At that time, some models (e.g. the immunologic and metabolomic models) recovered towards a state similar to a non-pregnant state, while others more closely reflected an early pregnant state or remain stable after delivery. This finding indicates that not all biological factors involved in pregnancy recover at a similar rates (Fig. 2D).

### 3.3 Stacked generalization

**Fig. 3.**
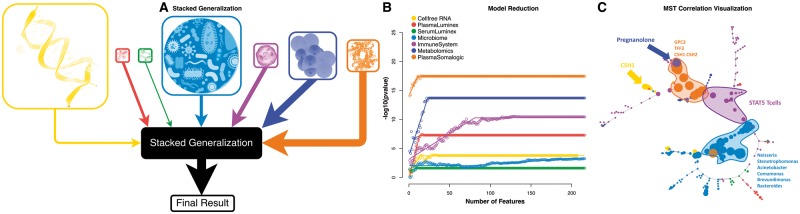
(**A**) Stacked generalization analysis. The size of the boxes is proportional to the ${\;\text{log}\;}_{1}0$ of the number of measurements in each dataset. The thickness of the arrow is proportional to the $-{\;\text{log}\;}_{1}0$ of *P*-value of a correlation test for gestational age; (**B**) The number of model components (x-axis) versus the *P*-value of the Spearman correlation between each model and gestational age (y-axis). Lines represent the piece-wise regression fit for calculation of the number of features. (**C**) Visualization of the most predictive features in a correlation network. The size of each node is proportional to the univariate correlation between that feature and gestational age. Color represents the corresponding dataset

A stacked generalization strategy was used to combine the predictive powers of the different omics datasets as described in Wolpert (1992). As illustrated in Figure 3A, an EN model was first trained on each dataset. Then, the estimations of gestational age produced by the seven independent models were merged using an additional EN model. Cross-validation was synchronized across all layers to ensure predictions were made on samples that had not been used for optimizing model coefficients. The free parameters of the models, as calculated using the inner CV procedure (see Section 2), are visualized in Supplementary Figure S1.

**Fig. 4.**
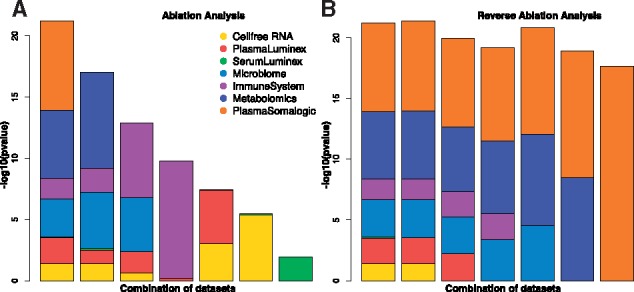
Ablation analysis to measure the collective predictive power of the model after removal of each dataset. At each iteration, the most (**A**) or least (**B**) important datasets were removed from stacked generalization. Color is proportional to the coefficients of the stacked generalization model. At each iteration, the algorithm was able to readjust the coefficients. This demonstrated that the algorithm could effectively use the remaining datasets to compensate for the latest removals

Ablation analysis, a procedure for investigating the path of dataset weights by iteratively retraining the stacked generalization model, was used to measure the relative contribution of each dataset to the final predictions (Fawcett and Hoos, 2016). This procedure was performed by iteratively removing the most important dataset from the mix (Fig. 4A). Importantly, for each iteration, the algorithm was able to recalculate new weights for the remaining datasets to partially compensate for any lost information. For example, after removal of the proteomic and metabolomic datasets, the algorithm significantly increased the weight of the predictions based on the immune system to compensate for the two removed datasets. Similar analysis in reverse order (Fig. 4B) revealed a minimal decrease in the predictive power when the most important dataset was preserved.

**Fig. 5.**
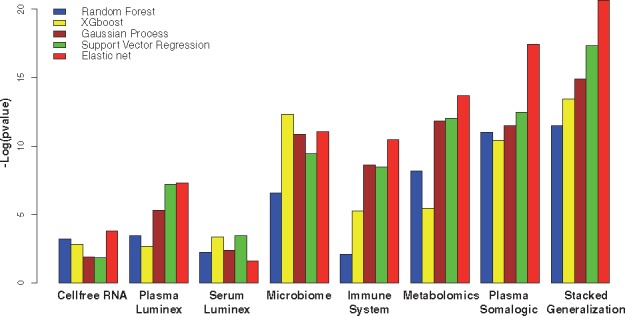
Empirical evaluation of elastic-net, random forest, XGboost, Gaussian Process and Support Vector Regression on each dataset, and the combination of all datasets. The hyper parameters of each method were tuned by the same two-layer leave-one-patient-out CV procedure for the prediction of gestational age on the test set. EN predominantly outperformed the other methods on most datasets, followed by support vector regression. XGboost outperformed the other algorithms on the microbiome dataset

To enable biological exploration, the top hits from each model were extracted using a bootstrapping strategy for sensitivity analysis (see Section 2 for details) and visualized using a minimum spanning tree of Spearman correlations between the selected features on a Fruchterman-Reingold layout (Fruchterman and Reingold, 1991), in Figure 3B and C, respectively. This resulted in a set of 226 interrelated features (Supplementary Table S1), revealing statistically robust interactions within and between each omics dataset. A Minimum Spanning Tree (MST) representation organized these interactions into a branched structure in which the distance between two features is proportional to the strength of the correlation between them. Metabolomics, transcriptomics and proteomics features primarily segregated into three clusters (Fig. 3C). Cell-based features from the immune system were distributed across the MST graph, forming a link between other omics datasets rather than being confined to a single cluster. The MST graph highlighted the connectivity between biological processes measured in the plasma (metabolomic, transcriptomic and proteomic measurements) or local compartments (microbiomic data) and cell-specific immune responses measured in the peripheral blood compartment.

### 3.4 Biological hypothesis generation

Several biologically plausible and hypothesis generating correlations between omics datasets emerged. Here, we highlight three of these data-driven hypotheses. In one instance, we illustrate how the integrative dataset can inform additional experiments that allow further exploration of the nature of observed interaction between different omics features.

With respect to the microbiomic data, a strong correlation was observed between changes in the composition of Neisseria bacterial species localized in the oral cavity as well as Bacteroides species in the gut and TCR$\gamma \delta^{+}$ T cells. This finding is consistent with the unique role of TCR$\gamma \delta^{+}$ T cells in mucosal immunity, particularly in the control of oral pathogens (Chien *et al.*, 2014; Moutsopoulos and Konkel, 2018; Wu *et al.*, 2014). Given increasing epidemiological evidence linking oral cavity dysbiosis and pregnancy-related complications, such as preterm labor and preeclampsia (Bassani *et al.*, 2007; Boggess *et al.*, 2003; Bošnjak *et al.*, 2006; Hajishengallis, 2015; Herrera *et al.*, 2007; Nabet *et al.*, 2010), our results raise the hypothesis that the correlation between the changes in oral bacterial species and TCR$\gamma \delta^{+}$ T cell frequencies may be disrupted in pathological pregnancies, such as preterm pregnancies.

With respect to the metabolomics dataset, the model revealed strong correlations between the plasma factor pregnanolone sulfate and the NF-*κ*B signaling in myeloid dendritic cells (mDCs) and regulatory T cells (Tregs). Pregnanolone sulfate, or 3α,5β-tetrahydroprogesterone(3α,5β-THP), is an endogenous steroid biosynthesized from progesterone. Modulation of immune cell function by progesterone and its derivative is well established (Druckmann and Druckmann, 2005). However, their roles in regulating the function of specific immune cell subsets during pregnancy are not fully understood. The results thus generated a novel hypothesis that pregnanolone sulfate may regulate important aspects of mDC and Treg functions during pregnancy.

With respect to the proteomic dataset, a three-way interaction between the transcriptomic, proteomic and cytomic datasets was particularly interesting, as it highlighted a novel connection between previously reported models of molecular clocks of pregnancy. This interaction contained the Chorionic Somatomammotropin Hormone-1 (CSH-1), represented at the transcript (cell-free RNA dataset) and protein (Somalogic dataset) levels, and the endogenous activity of the transcription factor STAT5 measured at the single-cell level in CD4+ and CD8+ T cell subsets. CSH-1 is known to bind to the prolactin receptor (Walsh and Kossiakoff, 2006), which signals through the JAK2/STAT5 signaling pathway (Gouilleux *et al.*, 1994). As such, results from the integrative analysis informs a novel hypothesis that CSH-1 may directly activate the JAK2/STAT5 signaling pathway in CD4^+^ and CD8^+^ T cell subsets during pregnancy.

The strong correlation observed between CSH-1 RNA and protein levels, and STAT5 activity in T cells (R = 0.59, *P* = $4.40\times 10^{-06}$) prompted further examination of this hypothesis in an in vitro model to determine whether CSH-1 can directly activate the JAK2/STAT5 signaling pathway in T cells. However, incubation of whole blood samples from non-pregnant or pregnant (Supplementary Fig. S3) women with CSH-1 did not induce the phosphorylation of STAT5 in CD4+ or CD8+ T cell subsets. On further inspection of the proteomic dataset, CSH-1 was found to belong to a community of tightly correlated plasma factors known to regulate the JAK/STAT signaling pathway. This community included the inflammatory cytokine Interleukin-2. Supplementary Figure S3 shows that, in contrast to CSH-1 or prolactin, incubation of whole blood samples with IL-2 induced a robust STAT5 phosphorylation signal in all major T cell subsets. These results suggested that in the context of pregnancy, the progressive increase in intracellular STAT5 activity in T cell subsets is likely driven by changes in IL-2 rather than CSH-1.

## 4 Discussion

We have described an analysis of seven high-throughput biological modalities during term pregnancy. An agnostic machine learning approach was used to evaluate the predictive power of each dataset for estimation of gestational age using biological signals. An additional machine learning layer was used to combine these estimations to further increase predictive power. Importantly, these datasets differed in both size and modularity. By taking this two layer approach, we prevented higher-dimensional datasets from overwhelming the final model. This both increased predictive power and facilitated biological interpretation.

Using this approach, we estimated the gestational age of the fetus at the time of each sampling. The stacked generalization algorithm produced models more accurate than models derived from any individual dataset. Ablation analysis (Fawcett and Hoos, 2016) was used to study the impact of each dataset on the final predictions. Importantly, this analysis showed that by retraining the stacked generalization model, other datasets could partially compensate for the removal of a given dataset. Using sensitivity analysis and piece-wise regression and sequential feature-reduction, each model was reduced to a limited number of required measurements. These were then used for correlation analysis, visualization and biological interpretation. These two complementary model reduction procedures lay the foundation for objective analysis to strike a balance between predictive-power and assay/sampling costs in resource-poor settings (e.g. a more expensive assay which requires a larger sample size from a complex biopsy may be replaceable by two cheaper and more feasible assays).

The study provided an integrated biological model of maternal changes during pregnancy, highlighting the interconnectivity of multiple biological systems. Notably, strong correlations between metabolomic, proteomic, transcriptomic features and specific immune cell signaling responses pointed at biologically plausible interactions. For example, the model identified a strong relationship between the steroid hormone pregnanolone sulfate and the signaling behavior of mDCs and Tregs. mDCs and Tregs play a critical role in feto-maternal tolerance and the maintenance of pregnancy (Aluvihare *et al.*, 2004; Erlebacher, 2013). Our data provide the basis for a novel hypothesis that pregnanolone sulfate plays a role in regulation of the function of these two cell types during pregnancy. Alternatively, recent evidence indicating that T cells can produce pregnenolone, the precursor of pregnanolone sulfate (Mahata *et al.*, 2014), suggests that immune cells may be a cellular source of pregnanolone sulfate production, providing another hypothesis for the observed correlations.

The study also shows that the biological interpretation of observed interactions between two model components benefits from exploring the communities of features that strongly correlate with these model components. As such, the integrative model revealed a strong interaction between the protein factor CSH-1 and STAT5 activity in CD4+ T cells. However, a community of protein factors correlating with CSH-1 contained the cytokine IL-2, a canonical activator of the JAK/STAT5 signaling pathway in CD4+ T cells (Mahmud *et al.*, 2013). Together with our in vitro data showing that stimulation with IL-2, but not with CSH-1, results in STAT5 phosphorylation in CD4+ T cells, these findings suggest that the interaction between CSH-1 and STAT5 activity in CD4+ T cells is likely indirectly mediated by IL-2. For example, activation of the PRL/CSH-1 receptor in cells other than T lymphocytes has been shown to promote the transcription of IL-2 (Sun *et al.*, 2004). CSH-1 may thus be implicated in the paracrine regulation of T cell function through positive regulation of IL-2 gene expression in other immune or non-immune cell types. When applied to postpartum samples collected 6 weeks after delivery, these models demonstrated that different biological modalities return to a non-pregnant state at different rates, reflecting synchronized pacemakers (Diemert and Arck, 2018). This finding motivates detailed biological analysis of the role of the inter-pregnancy interval (Girsen *et al.*, 2018) and history of preterm birth in adverse outcomes (Gaudillière *et al.*, 2015).

Selecting the hyperparameters of an EN model is largely a balancing act between sparsity and accuracy. In complex biological datasets, this is often confounded by the intrinsic characteristics of data including size and modularity (Waldmann *et al.*, 2013). To address this, a two-step CV procedure was used in this analysis. The inner layer enables optimization for the free-parameters of the EN model using an exhaustive grid search (Supplementary Fig. S1). The outer layer ensures the generalizability of the results to previously unseen samples. To increase sample size, each sample extracted at a trimester from a single subject was treated as an independent data point. To ensure the models were not biased by the dependency between samples donated by the same subject, all three trimesters of a given subject were excluded together in the same CV fold. Therefore, reported results are based on models that had access to no samples from a subject in the test-set. The samples used for testing purposes in all CV steps were synchronized across all models. Therefore, all test-set results (including those of the stacked generalization models) are reported only on samples that were blinded in all previous analyses.

This study has several limitations that have inspired our future plans. First, the number of subjects in this ‘proof-of-concept’ cohort was small relative to the number of measurements. In addition, recruitment from a single-care center limited the diversity of the dataset. Despite this, we were able to capture the chronology of biological changes during pregnancy. This correlation was not driven by age, BMI, or parity (partial correlation test *P *>* *0.05). However, given the racial disparities in pregnancy outcomes, replicating this analysis in more diverse cohorts is crucial. The March of Dimes Prematurity Center at Stanford University has already engaged in several international collaborations to directly address this. Similarly, the number of measurements was significantly larger than the cohort size, which increased the possibility of false positives. In addition to carefully designed cross-validation, feature reduction and clustering (e.g. Bien and Tibshirani, 2011) can be used to improve the predictive power of multivariate models in high-dimensional settings and enable exploration of more interactions between different datasets. These various approaches should be tested in an unbiased and collaborative setting (e.g. Aghaeepour *et al.*, 2016; Stolovitzky *et al.*, 2007) as large multiomics datasets become available. Finally, the current dataset included only one sample per trimester, and these samples were treated as independent datapoints. In the future, high-resolution sampling together with mixed effect models (Gałecki and Burzykowski, 2013) will combine the information content of different timepoints to produce increasingly more accurate prediction of pregnancy related events using serial sampling throughout pregnancy.

In summary, our study revealed a chronology of biologically-diverse events over the course of pregnancy. Our findings were enabled using seven high-throughput longitudinal biological assays of the same patient cohort. The computational pipeline introduced in this article can increase predictive power by combining datasets of various sizes and modularities in a balanced way. We expect this pipeline to be applicable to a wide range of studies beyond the field of pregnancy. Similarly, the dataset produced here provides a unique resource for future biological investigations. Particularly, this study can be used as a resource to identify correlates of any other features from one of the seven datasets that may be identified in future studies. Finally, by characterizing the biological chronology of normal pregnancy, this study provides the conceptual and analytical framework to analyze the complex interplays between various biological modalities that govern preterm birth and other pregnancy-related pathologies.

## Supplementary Material

Supplementary Data
