# Supplementary material for: Multiomics modeling of the immunome, transcriptome, microbiome, proteome and metabolome adaptations during human pregnancy
Source: Bioinformatics. 2018 Jul 2;35(1):95–103. doi: 10.1093/bioinformatics/bty537 (PMC6298056; doi:10.1093/bioinformatics/bty537)
Supplement: Supplementary Data [file bty537_supplemental-information.pdf]

Supplemental Information - Multiomics Modeling of the Immunome, Transcriptome, Microbiome, Proteome, and Metabolome Adaptations During Human Pregnancy

June 22, 2018

Contents

1 Supplemental Figures **2**

2 Supplemental Tables **7**

3 Biological Assays **13**

3.1 Cell-free RNA Transcriptome 13
3.2 Proteome 13
3.3 Microbiome 14
3.4 Immunome 15
3.5 Untargeted Metabolome 16

1 Supplemental Figures

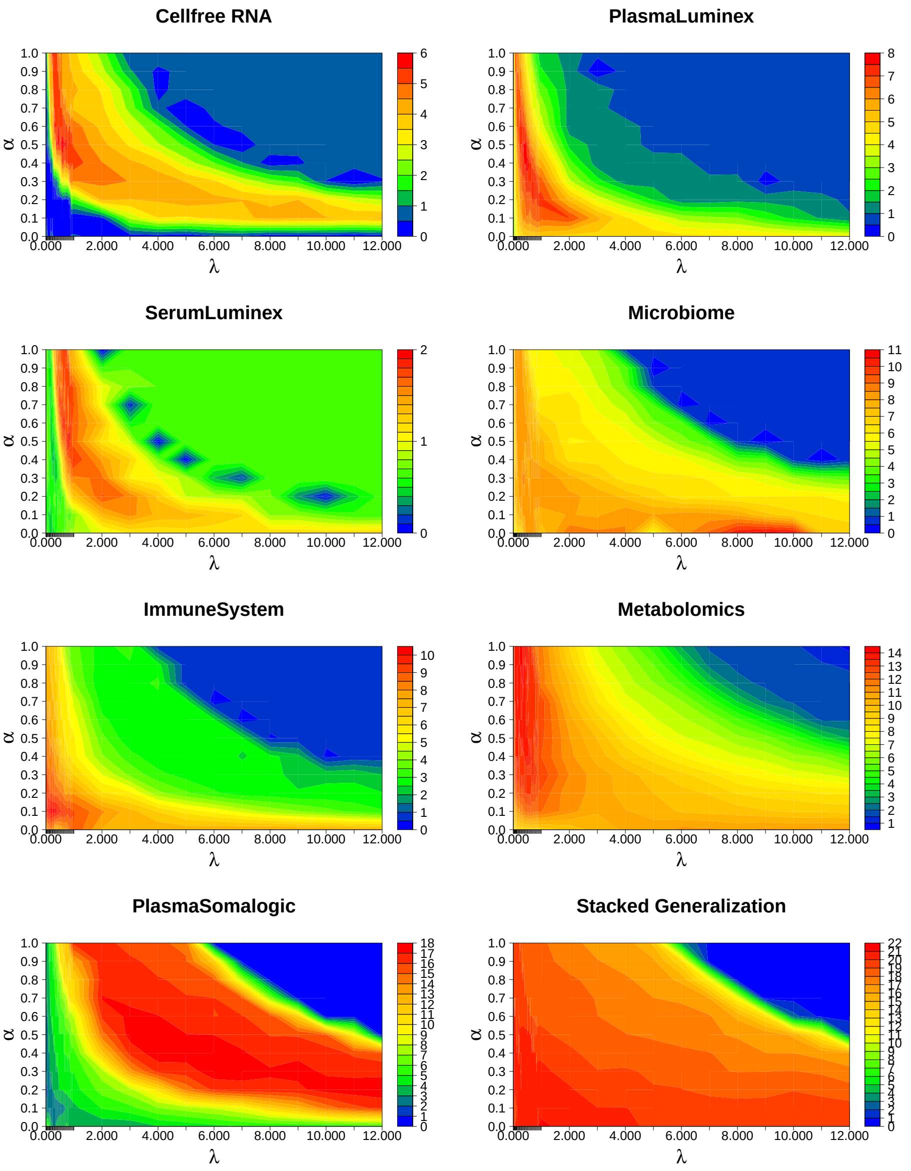

Fig. S1. Optimization of the λ and α hyper-parameters of EN. Distribution of $-\log_{10}$ of pvalue over $\lambda \in [0, 12]$ (x-axis), and $\alpha \in [0, 1]$ (y-axis), measured using leave-one-patient-out cross-validation, is visualized for seven individual and one stacked generalization model.

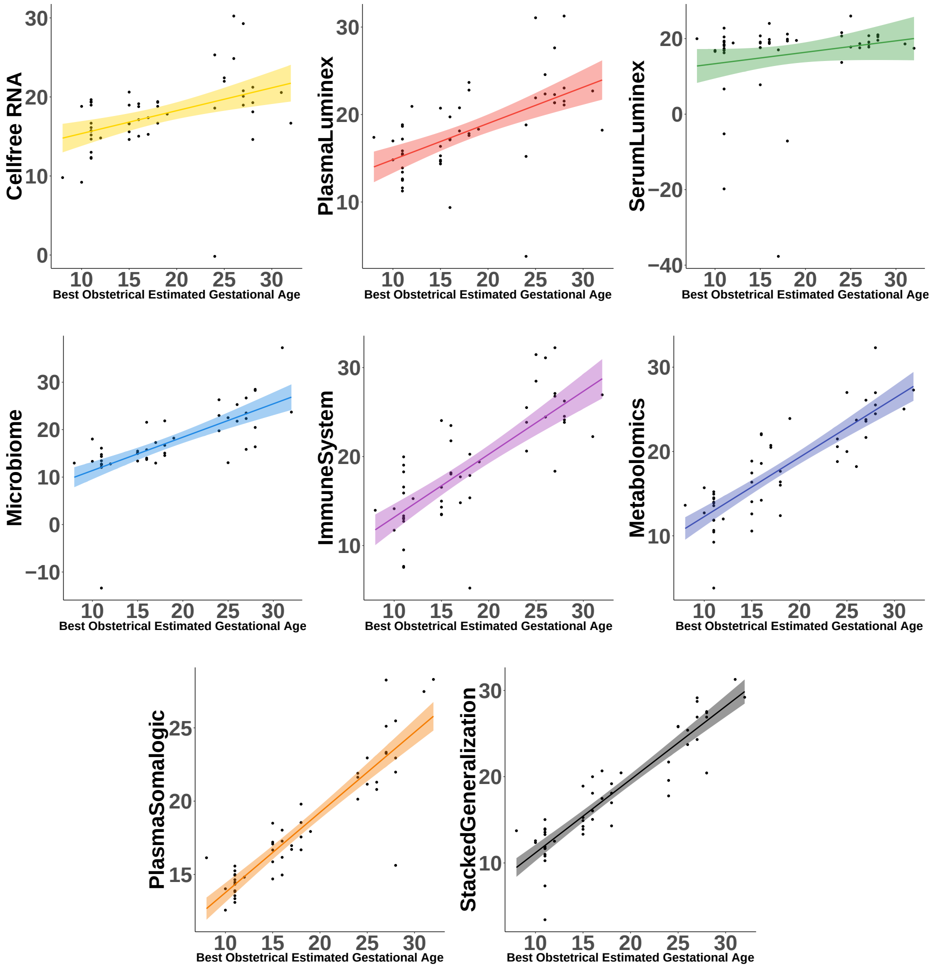

Fig. S2. Univariate regression analysis (and 95% confidence interval) between best obstetrical estimated gestational age (x-axis) and estimated gestational age from each model.

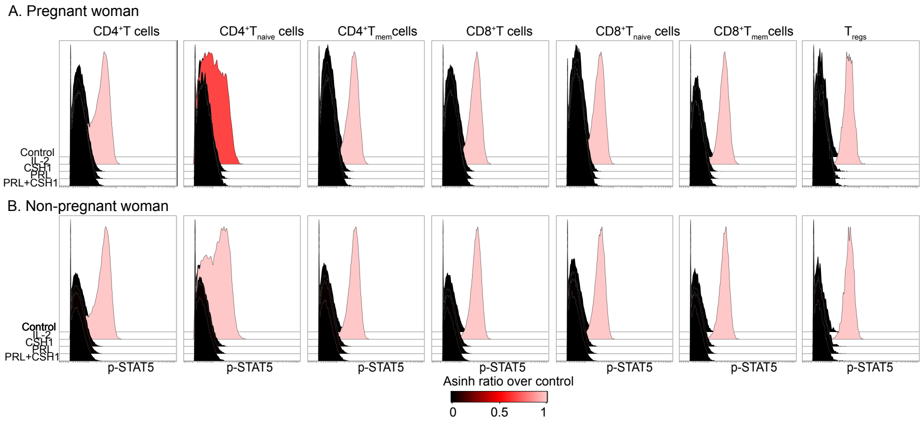

Fig. S3. Induction of STAT5 phosphorylation in T cell subsets stimulated with CSH1, PRL or IL-2. Whole blood samples from third trimester pregnant (A) or non-pregnant (B) women were left stimulated with PBS (control) or IL-2, CSH1, PRL or a combination of CSH1 and PRL. IL-2, but not CSH1 or PRL, induced STAT5 phosphorylation in all major T cell subsets. Representative histogram of three independent experiments are shown. Color scheme is proportional to the Asinh ratio of the p-STAT5 signal in stimulated vs. unstimulated conditions.

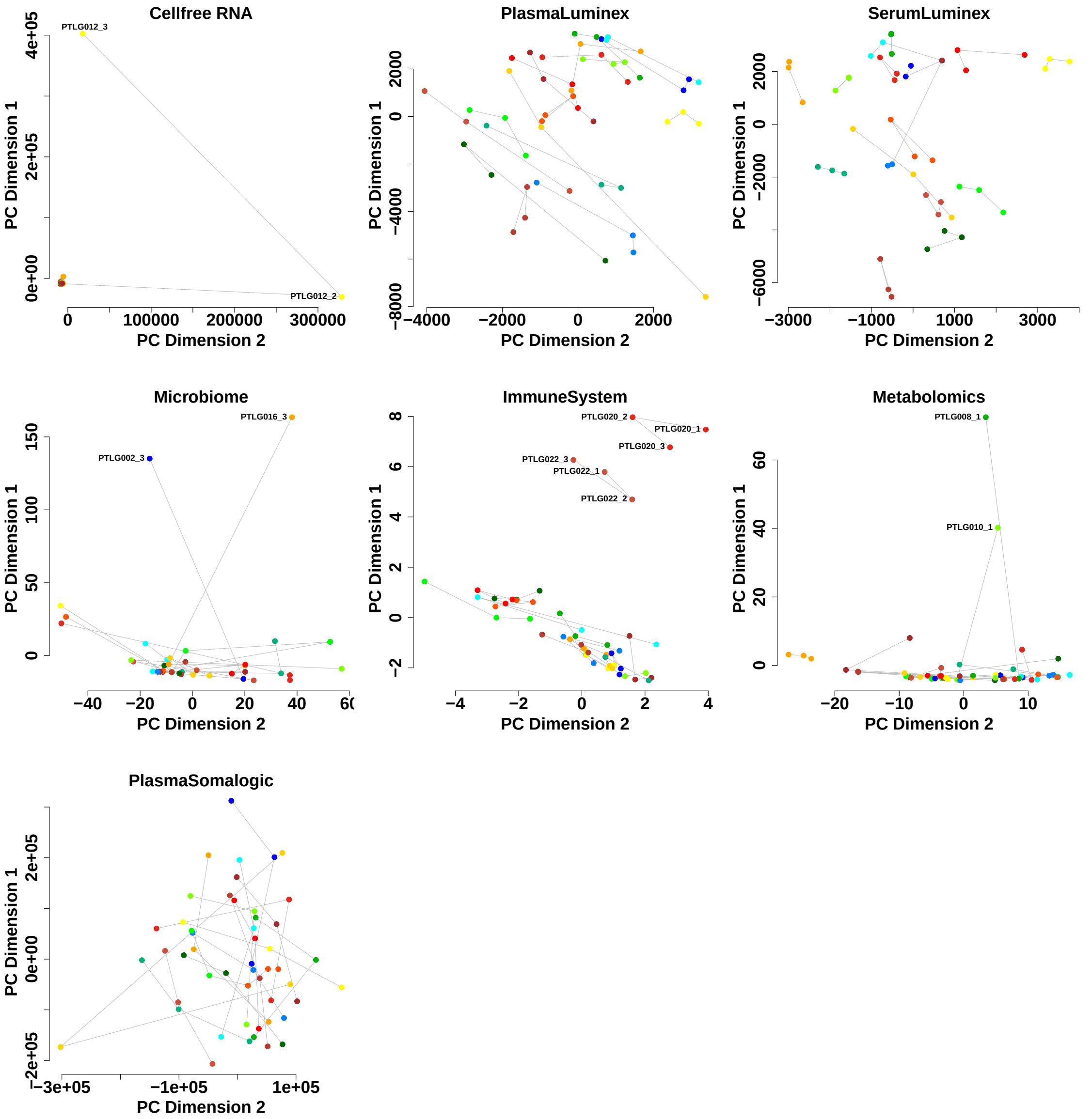

Fig. S4. The first two principal components of each dataset, colored by patient ID. Lines represent samples donated by the same individual. Labels are provided for outlier patients and the respective trimesters (4=postpartum).

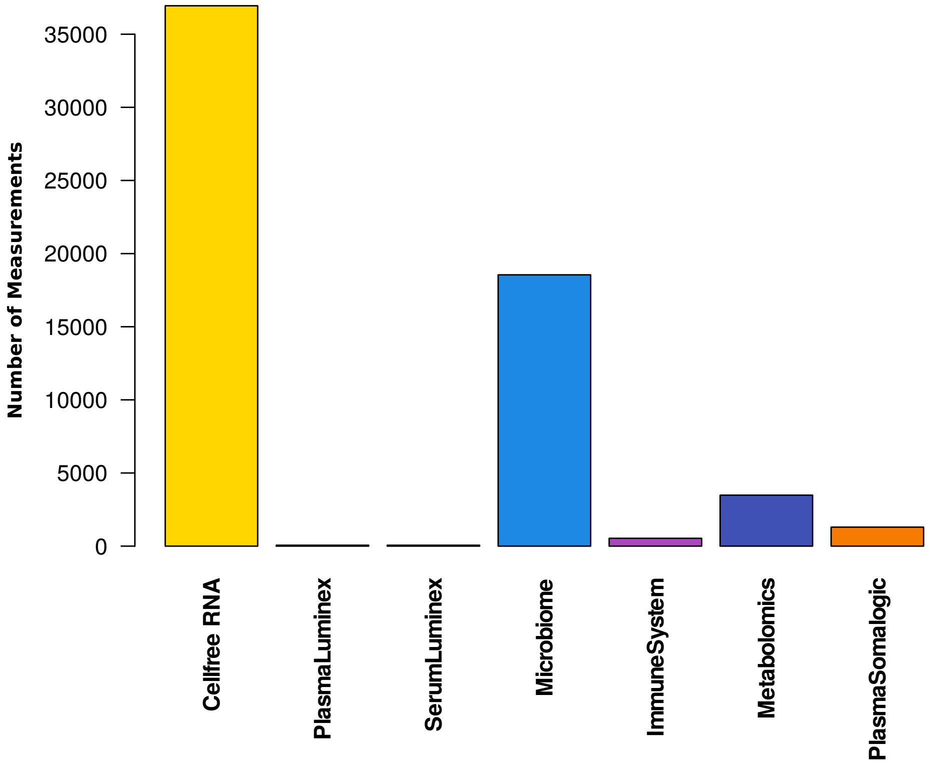

Fig. S5. Number of measurements (similar to Fig. 1B - without a log scale).

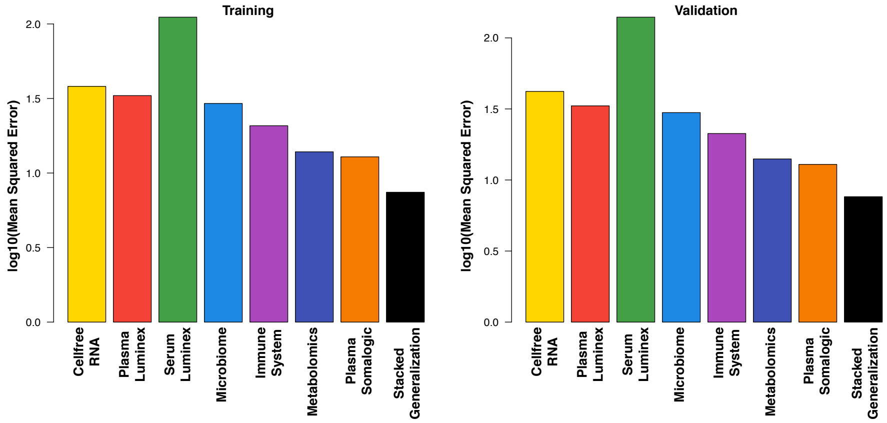

Fig. S6. Mean Squared Error (MSE) of the final models on each dataset individually and through stacked-generalization. $MSE = 1/n \sum_{i=1}^{n} (y_i - \hat{y}_i)^2$ where y is gestational age and $\hat{y}$ is the cross-validated estimation.

2 Supplemental Tables

Table S1: Selected features from each dataset. Colors are matched to Figure 1.

Features	p-value
HAGH	9.84e-05
HIST1H3I	3.20e-02
NCL	5.13e-03
HIST1H2AJ	2.90e-03
NDUFA5P11	3.98e-04
FAM200B	2.93e-02
RNA5SP150	1.96e-02
FHDC1	1.19e-02
CTB-36H16.2	4.12e-02
TRNP1	6.29e-02
CSH1	4.48e-05
EEF1A1P24	4.91e-04
RMDN2	6.15e-03
plasma-TGFA	8.26e-03
plasma-SDF1A	7.83e-01
plasma-IP10	9.34e-01
plasma-EOTAXIN	2.09e-02
plasma-IL12P40	1.11e-01
plasma-IL18	2.14e-02
plasma-MIG	2.49e-01
plasma-RESISTIN	3.56e-01
plasma-IL22	2.77e-01
plasma-PDGFBB	3.69e-01
plasma-VEGF	5.81e-02
plasma-PAI1	7.72e-03
plasma-ENA78	7.74e-01
serum-IL1B	5.96e-01
serum-IFNB	7.46e-01
serum-EOTAXIN	3.11e-02
serum-RANTES	6.27e-01
serum-TNFA	5.37e-01
serum-EGF	1.54e-01
serum-MIG	3.12e-01
serum-VCAM1	2.02e-02
serum-VEGF	8.00e-01
VaginalSwab_Lactobacillus	7.31e-01
Stool_Lactobacillus	5.64e-01
Saliva_Lactobacillus	1.07e-01
ToothGum_Lactobacillus	8.57e-02
VaginalSwab_Lactobacillus	7.82e-02
Saliva_Lactobacillus	1.62e-02
ToothGum_Lactobacillus	1.05e-01
Stool_Lactobacillus	8.90e-02

VaginalSwab_Staphylococcus	4.15e-03
ToothGum_Staphylococcus	1.58e-02
Stool_Staphylococcus	7.78e-01
VaginalSwab_Streptococcus	1.28e-01
Saliva_Streptococcus	1.40e-02
ToothGum_Streptococcus	3.98e-02
Stool_Streptococcus	1.18e-05
VaginalSwab_Bacteroides	1.20e-02
Saliva_Bacteroides	5.21e-05
ToothGum_Bacteroides	8.40e-04
Stool_Bacteroides	1.46e-07
ToothGum_Porphyromonas	6.18e-01
VaginalSwab_Anaerococcus	2.31e-01
Saliva_Anaerococcus	9.16e-01
ToothGum_Anaerococcus	4.13e-01
ToothGum_Varibaculum	9.01e-02
VaginalSwab_NA	6.71e-04
Saliva_NA	3.93e-04
ToothGum_NA	3.93e-04
Stool_NA	1.71e-02
Saliva_Lactobacillus	3.88e-03
ToothGum_Lactobacillus	1.01e-03
Stool_Lactobacillus	1.28e-01
ToothGum_Prevotella_6	7.62e-01
Stool_Prevotella_6	5.20e-05
VaginalSwab_NA	6.80e-07
VaginalSwab_Pseudomonas	6.40e-01
ToothGum_Pseudomonas	4.60e-01
Stool_Pseudomonas	6.34e-01
Saliva_[Eubacterium]_ventriosum_group	1.52e-08
ToothGum_[Eubacterium]_ventriosum_group	1.52e-08
VaginalSwab_Bifidobacterium	6.04e-03
VaginalSwab_Acinetobacter	7.43e-02
Saliva_Acinetobacter	4.71e-02
ToothGum_Acinetobacter	2.09e-01
Stool_Acinetobacter	3.33e-05
VaginalSwab_NA	4.51e-03
Saliva_NA	7.84e-04
ToothGum_NA	8.66e-05
Stool_NA	4.90e-06
VaginalSwab_Yersinia	3.13e-02
Saliva_Yersinia	8.37e-02
ToothGum_Yersinia	1.43e-01
Stool_Yersinia	5.20e-03
VaginalSwab_Acinetobacter	1.58e-03
Saliva_Acinetobacter	1.49e-04

ToothGum_Acinetobacter	5.67e-04
Stool_Acinetobacter	9.09e-03
ToothGum_Dialister	1.48e-01
Stool_Dialister	8.30e-05
VaginalSwab_NA	6.80e-07
VaginalSwab_Acinetobacter	6.92e-01
Saliva_Acinetobacter	5.09e-01
ToothGum_Acinetobacter	3.70e-01
Stool_Acinetobacter	8.57e-03
Saliva_Neisseria	3.58e-06
ToothGum_Neisseria	3.58e-06
VaginalSwab_Stenotrophomonas	6.80e-07
VaginalSwab_Acinetobacter	6.80e-07
VaginalSwab_Herbaspirillum	1.47e-01
Saliva_Sphingobium	2.22e-02
ToothGum_Sphingobium	1.97e-01
VaginalSwab_Brevundimonas	6.80e-07
VaginalSwab_NA	6.80e-07
VaginalSwab_Anaerofilum	2.96e-05
Saliva_Anaerofilum	3.86e-07
ToothGum_Anaerofilum	8.29e-08
VaginalSwab_Methylobacillus	1.60e-04
VaginalSwab_Comamonas	1.23e-06
Bcells	2.50e-01
CD16+CD56-NKcells	3.49e-01
CD4+Tcells_mem	2.87e-01
CD4+Tcells_naive	2.85e-01
CD45RA+Tregs	2.88e-01
CD7+NKcells	1.41e-01
cMCs	2.85e-01
Gr	6.29e-02
intMCs	7.53e-01
ncMCs	7.64e-01
Tbet+CD4+Tcells_naive	5.37e-01
TCRgd+Tcells	4.28e-01
CD16+CD56-NKcells_STAT1_IFNa100	4.86e-04
CD45RA-Tregs_STAT1_IFNa100	5.30e-02
CD7+NKcells_STAT1_IFNa100	4.92e-04
CD16+CD56-NKcells_STAT5_IFNa100	6.88e-01
CD4+Tcells_naive_STAT5_IFNa100	8.08e-02
CD4+Tcells_STAT5_IFNa100	6.02e-02
CD56+CD16-NKcells_STAT5_IFNa100	5.12e-01
CD7+NKcells_STAT5_IFNa100	6.08e-01
TCRgd+Tcells_STAT5_IFNa100	7.69e-02
Gr_ERK_IL100	8.96e-01
mDCs_ERK_IL100	4.84e-01

Tbet+CD4+Tcells_naive_ERK_IL100	3.53e-01
Gr_STAT5_IL100	4.86e-04
ncMCs_STAT5_IL100	1.06e-01
CD16+CD56-NKcells_CREB_LPS100	1.39e-01
CD56+CD16-NKcells_CREB_LPS100	2.15e-03
CD7+NKcells_CREB_LPS100	1.41e-02
mDCs_ERK_LPS100	3.47e-02
CD45RA-Tregs_IkB_LPS100	2.16e-02
mDCs_IkB_LPS100	8.79e-02
Tregs_IkB_LPS100	2.34e-02
mDCs_NFkB_LPS100	5.31e-04
CD56+CD16-NKcells_S6_LPS100	1.61e-02
Tregs_S6_LPS100	5.30e-01
CD45RA-Tregs_CREB_Unstim	7.53e-01
intMCs_CREB_Unstim	8.96e-01
ncMCs_CREB_Unstim	3.04e-01
pDCs_CREB_Unstim	4.24e-01
Tbet+CD8+Tcells_naive_CREB_Unstim	9.42e-01
TCRgd+Tcells_CREB_Unstim	3.42e-01
Tregs_CREB_Unstim	7.57e-01
CD45RA-Tregs_ERK_Unstim	5.64e-01
ncMCs_ERK_Unstim	5.47e-01
pDCs_ERK_Unstim	4.32e-01
Tregs_ERK_Unstim	5.07e-01
Bcells_IkB_Unstim	3.81e-01
CD4+Tcells_naive_IkB_Unstim	1.18e-01
CD4+Tcells_IkB_Unstim	1.02e-01
CD8+Tcells_naive_IkB_Unstim	5.84e-02
CD8+Tcells_IkB_Unstim	2.28e-01
intMCs_IkB_Unstim	1.27e-01
Tbet+CD8+Tcells_naive_IkB_Unstim	8.16e-01
M-MDSC_MAPKAPK2_Unstim	6.11e-01
CD4+Tcells_naive_NFkB_Unstim	1.28e-01
CD4+Tcells_NFkB_Unstim	1.76e-01
CD8+Tcells_naive_NFkB_Unstim	2.17e-01
Tbet+CD8+Tcells_mem_NFkB_Unstim	5.00e-01
Bcells_S6_Unstim	8.49e-01
CD4+Tcells_S6_Unstim	8.72e-02
CD45RA-Tregs_S6_Unstim	2.72e-01
CD8+Tcells_mem_S6_Unstim	5.29e-01
intMCs_S6_Unstim	1.82e-01
Tbet+CD8+Tcells_mem_S6_Unstim	5.16e-01
Tbet+CD8+Tcells_naive_S6_Unstim	5.22e-01
TCRgd+Tcells_S6_Unstim	1.19e-01
Gr_STAT1_Unstim	8.17e-01
Tbet+CD4+Tcells_naive_STAT1_Unstim	1.73e-01

CD4+Tcells_naive_STAT3_Unstim	3.51e-01
CD45RA+Tregs_STAT3_Unstim	6.71e-01
CD4+Tcells_mem_STAT5_Unstim	1.70e-03
CD4+Tcells_naive_STAT5_Unstim	3.35e-05
CD4+Tcells_STAT5_Unstim	3.96e-04
CD45RA+Tregs_STAT5_Unstim	2.56e-02
CD8+Tcells_naive_STAT5_Unstim	8.73e-04
CD8+Tcells_STAT5_Unstim	1.82e-03
pDCs_STAT5_Unstim	7.66e-01
Tbet+CD4+Tcells_mem_STAT5_Unstim	1.29e-02
Tregs_STAT5_Unstim	2.28e-02
Creatinine	6.24e-02
Hypoxanthine	3.09e-03
PE(16:0/22:6(4Z,7Z,10Z,13Z,16Z,19Z))	3.24e-03
Benzenearsonic acid	2.77e-01
PE(14:0/20:3(5Z,8Z,11Z))	2.79e-02
PC(16:0/22:4(7Z,10Z,13Z,16Z))	4.16e-01
PC(14:0/20:2(11Z,14Z))	7.69e-03
Biliverdin	1.01e-01
Hydroxyzileuton	7.09e-03
L-Acetylcarnitine	5.27e-02
Edetic Acid	9.91e-01
2-Methyl-3-ketovaleric acid	2.77e-01
2-Methyl-3-ketovaleric acid	1.16e-02
Ethyl glucuronide	1.63e-04
4-[3-(4-FLUOROPHENYL)-1H-PYRAZOL-4-YL]PYRIDIN	2.76e-02
Sodium ferulate	8.07e-03
3-(1,1-dioxido-4H-1,2,4-benzothiadiazin-3-yl)	1.26e-01
1-Kestose	8.73e-03
Edetic Acid	4.61e-01
Edetic Acid	9.08e-02
LysoPC(18:2(9Z,12Z))	3.12e-03
Sunitinib	3.73e-11
Oleic acid	3.16e-01
CSH1 CSH2	3.23e-15
CSF1R	3.80e-11
IGFBP7	1.90e-09
BMP1	7.07e-10
LGMN	7.08e-09
PRSS27	9.83e-08
TFF3	1.96e-15
GPC3	1.24e-16
GRN	1.77e-11
AFP	6.78e-12
SST	1.65e-11

3 Biological Assays

3.1 Cell-free RNA Transcriptome

Cell-free RNA (CfRNA) was extracted from 1 mL of plasma using Plasma/Serum Circulating RNA and Exosomal Purification kit (Norgen, cat 42800) following manufacture instruction. The residue of DNA was digested using Baseline-ZERO DNase (Epicentre) and then cleaned by RNA Clean and Concentrator-5 kit (Zymo). RNA was eluted to 12 ul in elution buffer.

One half of the eluted RNA was used for sequencing library preparation using SMARTer Stranded Total RNAseq-Pico Input Mammalian kit (Clontech) according to the manufacturer's manual. Short read sequencing was performed using the Illumina NextSeq (2×75 bp) platform to the depth of more than 10 million reads per samples. The sequencing reads were mapped to human reference genome (hg38) using STAR aligner. Duplicates were removed by Picard and then unique reads were quantified using htseq-count.

3.2 Proteome

Blood was collected into EDTA tubes, put on ice, centrifuged within 60 minutes, and plasma was stored at $-80°C$ until further processing. A first analysis was performed in the Human Immune Monitoring Center (HIMC) at Stanford University using a standard, human 62-plex kit from eBiosciences/Affymetrix (San Diego, CA) according to the manufacturer's recommendations. The assay uses antibodies to quantify proteins over a 3 to 4 log dynamic range using color-coded beads pre-coated with analyte-specific capture antibodies and biotinylated detection antibodies to form an analyte specific antibody-antigen sandwich. Briefly, mixed antibody-linked beads were added to 96-well plates and washed. After an overnight incubation, plates were washed and biotinylated detection antibodies were added, plates were washed again, streptavidin-PE was added, plates were washed again, and finally reading buffer was added. Beads and respective analytes of duplicate samples were read on a dual-laser detection instrument (Luminex 200, Luminex, Friendswood, TX). Custom assay control beads by Radix Biosolutions (Georgetown, TX) were added to all wells. According to HIMC's quality control parameters, samples with a bead count < 50 and a coefficient of variation (CV) $> 30\%$ were excluded from analyses. A second analysis on the same samples was performed by SomaLogic, Inc. (Boulder, CO) using a highly-multiplex aptamer-based platform capturing $1,310$ proteins [Rohloff et al., 2014, Gold et al., 2010]. This assay quantifies proteins over a wide dynamic range (> 8 log) using chemically-modified aptamers with slow off-rate kinetics (SOMAmer reagents). Each SOMAmer reagent is a unique, high-affinity, single-strand DNA endowed with functional groups mimicking amino acid side chains. In brief, samples were incubated on 96-well plates with a mixture of SOMAmer reagents. Two sequential bead-based immobilization and washing steps were used to eliminate non-specifically bound proteins, unbound proteins, and unbound SOMAmer reagents from pro-

tein target-bound reagents. After eluting SOMAmer reagents from the target proteins, the fluorescently-labeled reagents were quantified on an Agilent hybridization array (Agilent Technologies, Santa Clara, CA). Data were normalized in four specific steps and according to assay data quality control procedures defined in the good laboratory practice quality system of SomaLogic, Inc. Normalization steps controlled for signal intensity biases introduced by differential hybridization efficiencies and the overall brightness of plates (accepted range 0.4 to 2.5), collection protocol artifacts (accepted range 0.4 to 2.5), and batch effects between different plates (accepted range 0.5 to 2.0).

3.3 Microbiome

Self-sampling of the vagina, gut, saliva, and tooth/gum was performed weekly by study participants. Sterile Catch-All^{TM} Sample Collection Swabs (Epicentre Biotechnologies, Madison, WI, USA) were used to obtain material from: 1) vagina (midvaginal wall), 2) gut (rectal mucosa), and 3) tooth/gum (molar tooth surfaces including along the gum-line). Saliva was collected at a volume of $2-5mL$ in a sterile $50mL$ conical collection tube (B-D Falcon, Franklin Lakes, NJ, USA). All clinical specimens were placed immediately after collection at $-20°$C until transport to the laboratory for storage at $-80°$C until further processing.

Whole genomic DNA was extracted from each vaginal swab by means of the PowerSoil DNA isolation kit (MO BIO Laboratories) according to the manufacturer's protocol except for the inclusion of a 10-min incubation at 65°C immediately after the addition of solution C1. The V4 hypervariable region of the 16S rRNA gene was amplified by PCR. The forward PCR primer (5′ AAT GAT ACG GCG ACC ACC GAG ATC TAC ACG CTN NNN NNN NNN NNT ATG GTA ATT GTG TGY CAG CMG CCG CGG TAA 3′) was a 75-nucleotide (nt) fusion primer consisting of the 32-nt Illumina adapter (designated by bold), a unique 12-nt barcode to label each amplicon (designated by the N's), a 10-nt forward primer pad, a 2-nt linker (GT), and the 19-nt broad-range bacterial primer 515F (designated by underlining). The 56-nt reverse primer (5' CAA GCA GAA GAC GGC ATA CGA GAT AGT CAG CCA GCC GGA CTA CNV GGG TWT CTA AT 3′) consisted of the 24-nt Illumina adapter (designated by bold), a 10-nt reverse primer pad, a 2-nt reverse primer linker (CC), and the 20-nt broad-range bacterial primer 806R (designated by underlining).

Triplicate 25-μL PCRs were carried out by using 1× HotMasterMix (5 PRIME), 0.4 μM concentrations of each commercially synthesized primer, and 3 μL of prepared DNA template. Thermal cycling conditions consisted of an initial denaturing step of 94°C for 3 min, followed by 30 cycles of 94°C for 45s, 50°C for 60s, and 72°C for 90s, with a final extension step of 72°C for 10 minutes. Upon completion of the PCRs, the corresponding triplicate reaction mixtures were pooled and purified by using the Ultra-clean-htp 96-well PCR clean-up kit (Mo Bio Laboratories) according to the manufacturer's protocol. DNA concentrations from each triplicate pool were quantified using the Quant-iT High-Sensitivity dsDNA Assay Kit (Invitrogen) and combined in equimolar

ratios into a single tube. The resulting amplicon mixture was concentrated by ethanol precipitation and resuspended in 100 μL of molecular biology-grade water (Life Technologies). The resuspended amplicon mixture was gel purified and recovered using a QIAquick gel extraction kit (Qiagen). Recovered PCR products were sequenced on an Illumina HiSeq 2500 instrument (Illumina) at the W. M. Keck Center for Comparative Functional Genomics at the University of Illinois, Urbana–Champaign, IL.

Bioinformatics processing largely followed the DADA2 Workflow for Big Data (`benjjneb.github.io/dada2/bigdata_paired.html`). Forward/reverse read pairs were trimmed and filtered, with forward reads truncated at 245 nt and reverse reads at 235 nt, no ambiguous bases allowed, and each read required to have less than two expected errors based on their quality scores. The relationship between quality scores and error rates was estimated for each sequencing run to reduce batch effects arising from run-to-run variability. ASVs were independently inferred from the forward and reverse of each sample using the run-specific error rates, and then read pairs were merged. Chimeras were identified in each sample, and ASVs were removed if identified as chimeric in a sufficient fraction of the samples in which they were present. Taxonomic assignment was performed against the Silva v123 database using the implementation of the RDP naive Bayesian classifier available in the dada2 `R` package [Wang et al., 2007, Quast et al., 2012]. Lactobacillus species were assigned by hand via BLAST against sequences from cultured Lactobacillus strains.

3.4 Immunome

Whole blood samples were stimulated for 15 min with either LPS, IFNα, a cocktail containing IL-2 and IL-6, or left unstimulated. Samples were then processed using a standardized protocol for fixation (SmartTube Inc), barcoding and antibody staining of whole blood samples for mass cytometry analysis [Bodenmiller et al., 2012]. For further details see Aghaeepour et al. [2017]. Three categories of immune features were derived for integrative analysis:

Cell frequency features: cell frequencies were expressed as a percentage of gated singlets in the case of neutrophils, and as a percentage of mononuclear cells (CD45+CD66−) in the case of all other cell types.

Endogenous signaling immune features: Endogenous intracellular signaling activities were derived from the analysis of unstimulated blood samples. The signal intensity of the following functional markers was simultaneously quantified per single cell: phospho (p) STAT1, pSTAT3, pSTAT5, pNFκB, total IκB, pMAPKAPK2, pP38, prpS6, pERK1/2, and pCREB. For each cell type, signaling immune features were calculated as the median signal intensity (arcsinh transformed value) of each signaling protein.

Intracellular signaling response features: the signal intensity of all functional markers was analyzed from samples stimulated with LPS, IFNα or IL. For each cell type, signaling responses were calculated as the difference in median signal intensity (arcsinh transformed value) of each signaling protein between the stimulated and unstimulated conditions.

3.5 Untargeted Metabolome

Metabolites were extracted from plasma and analyzed using a broad coverage untargeted metabolomics platform as described previously [Contrepois et al., 2015]. Briefly, metabolites were extracted using 1 : 1 : 1 acetone:acetonitrile:methanol, evaporated to dryness under nitrogen and reconstituted in 1:1 methanol:water before analysis. Metabolic extracts were analyzed four times using HILIC and RPLC separation in both positive and negative ionization modes ([Piening et al., 2018]). Data were acquired on a Thermo Q Exactive plus mass spectrometer for HILIC and a Thermo Q Exactive mass spectrometer for RPLC. Both instruments were equipped with a HESI-II probe and operated in full MS scan mode. MS/MS data were acquired on quality control samples (QC) consisting of an equimolar mixture of all the samples in the study. HILIC experiments were performed using a ZIC-HILIC column 2.1×100 mm, 3.5 μm, 200Å (Merck Millipore) and mobile phase solvents consisting of 10 mM ammonium acetate in 50/50 acetonitrile/water (A) and 10 mM ammonium acetate in 95/5 acetonitrile/water (B). RPLC experiments were performed using a Zorbax SBaq column 2.1×50 mm, 1.7 μm, 100Å (Agilent Technologies) and mobile phase solvents consisting of 0.06% acetic acid in water (A) and 0.06% acetic acid in methanol (B).

Metabolites were extracted and analyzed in a randomized order. Data generated from each separation and acquisition mode were analyzed independently using Progenesis QI (Nonlinear Dynamics). Features from blanks and that didn't present sufficient linearity upon serial dilution of QC samples were discarded. In addition, features with a median intensity value below 5E4 in HILIC mode and 1E4 in RPLC mode were discarded. Also, metabolic features present in less than 33% of the samples were removed from the analysis. Missing values were imputed using the k-nearest neighbors' method. Intra-batch variation was corrected using the LOESS normalization method on QC injected repetitively along the batch. In order to account for increased blood volume during pregnancy, metabolomics data were scaled using a Probabilistic Quotient transformation [Dieterle et al., 2006]. Metabolic features were putatively identified by matching the accurate masses ($\pm$ 5 ppm) against a local database containing $+60,000$ entries (compilation of various public databases such as HMDB, FoodB, DrugBank). $3,473$ metabolic features matched the database and were used for prediction. Metabolites of interest were validated manually by comparing the retention time and/or fragmentation spectrum to a local or public spectral libraries.

References

N. Aghaeepour, E. A. Ganio, D. Mcilwain, A. S. Tsai, M. Tingle, S. Van Gassen, D. K. Gaudilliere, Q. Baca, L. McNeil, R. Okada, et al. An immune clock of human pregnancy. *Science immunology*, 2(15):eaan2946, 2017.

B. Bodenmiller, E. R. Zunder, R. Finck, T. J. Chen, E. S. Savig, R. V. Bruggner,

E. F. Simonds, S. C. Bendall, K. Sachs, P. O. Krutzik, et al. Multiplexed mass cytometry profiling of cellular states perturbed by small-molecule regulators. *Nature biotechnology*, 30(9):858–867, 2012.

K. Contrepois, L. Jiang, and M. Snyder. optimized analytical procedures for the untargeted metabolomic profiling of human urine and plasma by combining hydrophilic interaction (hilic) and reverse-phase liquid chromatography (rplc)–mass spectrometry. *Molecular & Cellular Proteomics*, 14(6):1684–1695, 2015.

F. Dieterle, A. Ross, G. Schlotterbeck, and H. Senn. Probabilistic quotient normalization as robust method to account for dilution of complex biological mixtures. application in 1h nmr metabonomics. *Analytical chemistry*, 78(13): 4281–4290, 2006.

L. Gold, D. Ayers, J. Bertino, C. Bock, A. Bock, E. N. Brody, J. Carter, A. B. Dalby, B. E. Eaton, T. Fitzwater, et al. Aptamer-based multiplexed proteomic technology for biomarker discovery. *PloS one*, 5(12):e15004, 2010.

B. D. Piening, Z. Wenyu, C. Kévin, H. Röst, G. J. G. Urban, T. Mishra, B. M. Hanson, E. J. Bautista, S. Leopold, C. Y. Yeh, D. Spakowicz, I. Banerjee, C. Chen, K. Kukurba, D. Perelman, C. Craig, E. Colbert, D. Salins, S. Rego, S. Lee, C. Zhang, J. Wheeler, M. R. Sailani, L. Liang, C. Abbott, M. Gerstein, A. Mardinoglu, U. Smith, D. L. Rubin, S. Pitteri, E. Sodergren, T. L. McLaughlin, G. M. Weinstock, and M. P. Snyder. Integrative personal omics profiles during periods of weight gain and loss. *Cell Systems*, pages 1–14, 2018.

C. Quast, E. Pruesse, P. Yilmaz, J. Gerken, T. Schweer, P. Yarza, J. Peplies, and F. O. Glöckner. The silva ribosomal rna gene database project: improved data processing and web-based tools. *Nucleic acids research*, 41(D1):D590–D596, 2012.

J. C. Rohloff, A. D. Gelinas, T. C. Jarvis, U. A. Ochsner, D. J. Schneider, L. Gold, and N. Janjic. Nucleic acid ligands with protein-like side chains: modified aptamers and their use as diagnostic and therapeutic agents. *Molecular Therapy—Nucleic Acids*, 3(10):e201, 2014.

Q. Wang, G. M. Garrity, J. M. Tiedje, and J. R. Cole. Naive bayesian classifier for rapid assignment of rrna sequences into the new bacterial taxonomy. *Applied and environmental microbiology*, 73(16):5261–5267, 2007.
